# Axon guidance cue SEMA3A promotes the aggressive phenotype of basal-like PDAC

**DOI:** 10.1136/gutjnl-2023-329807

**Published:** 2024-04-26

**Authors:** Francesca Lupo, Francesco Pezzini, Davide Pasini, Elena Fiorini, Annalisa Adamo, Lisa Veghini, Michele Bevere, Cristina Frusteri, Pietro Delfino, Sabrina D'agosto, Silvia Andreani, Geny Piro, Antonia Malinova, Tian Wang, Francesco De Sanctis, Rita Teresa Lawlor, Chang-il Hwang, Carmine Carbone, Ivano Amelio, Peter Bailey, Vincenzo Bronte, David Tuveson, Aldo Scarpa, Stefano Ugel, Vincenzo Corbo

**Affiliations:** 1 Department of Engineering for Innovation Medicine, University of Verona, Verona, Italy; 2 Department of Medicine, University of Verona, Verona, Italy; 3 ARC-Net Research Centre, University of Verona, Verona, Italy; 4 Department of Diagnostic and Public Health, University of Verona, Verona, Italy; 5 Division of Immunology, Transplantation and Infectious Diseases, IRCSS San Raffaele, Milan, Italy; 6 Human Technopole, Milan, Italy; 7 Department of Biochemistry and Molecular Biology, University of Würzburg, Wurzburg, Germany; 8 Department of Medical and Surgical Sciences, Fondazione Policlinico Universitario Agostino Gemelli IRCCS, Roma, Italy; 9 Microbiology and Molecular Genetics, UC Davis Department of Microbiology, Davis, California, USA; 10 Division of Systems Toxicology, Department of Biology, University of Konstanz, Konstanz, Germany; 11 Wolfson Wohl Cancer Research Centre, University of Glasgow, Glasgow, UK; 12 Cold Spring Harbor Laboratory, Cold Spring Harbor, New York, USA

**Keywords:** PANCREATIC CANCER

## Abstract

**Objective:**

The dysregulation of the axon guidance pathway is common in pancreatic ductal adenocarcinoma (PDAC), yet our understanding of its biological relevance is limited. Here, we investigated the functional role of the axon guidance cue SEMA3A in supporting PDAC progression.

**Design:**

We integrated bulk and single-cell transcriptomic datasets of human PDAC with in situ hybridisation analyses of patients’ tissues to evaluate SEMA3A expression in molecular subtypes of PDAC. Gain and loss of function experiments in PDAC cell lines and organoids were performed to dissect how SEMA3A contributes to define a biologically aggressive phenotype.

**Results:**

In PDAC tissues, SEMA3A is expressed by stromal elements and selectively enriched in basal-like/squamous epithelial cells. Accordingly, expression of SEMA3A in PDAC cells is induced by both cell-intrinsic and cell-extrinsic determinants of the basal-like phenotype. *In vitro*, SEMA3A promotes cell migration as well as anoikis resistance. At the molecular level, these phenotypes are associated with increased focal adhesion kinase signalling through canonical SEMA3A-NRP1 axis. SEMA3A provides mouse PDAC cells with greater metastatic competence and favours intratumoural infiltration of tumour-associated macrophages and reduced density of T cells. Mechanistically, SEMA3A functions as chemoattractant for macrophages and skews their polarisation towards an M2-like phenotype. In SEMA3A^high^ tumours, depletion of macrophages results in greater intratumour infiltration by CD8+T cells and better control of the disease from antitumour treatment.

**Conclusions:**

Here, we show that SEMA3A is a stress-sensitive locus that promotes the malignant phenotype of basal-like PDAC through both cell-intrinsic and cell-extrinsic mechanisms.

**What is already known on this topic**

- PDAC cell states manifest as a wide range of environmentally contingent traits.
- The PDAC cell state that displays aggressive biological behavior underscores the dysregulation of axon guidance programs.
- Elevated tissue expression of SEMA3A is consistently associated with poor outcome in PDAC.

**What this study adds**

- SEMA3A is a stress-sensitive locus that responds to the different microenvironmental challenges of the complex PDAC tumour microenvironment.
- SEMA3A exerts both cell-autonomous and non-cell autonomous effects to sustain PDAC progression and drive resistance to chemotherapy.
- Tumour-derived SEMA3A favours intra-tumoral infiltration of macrophages and exclusion of T cells.

**How this study might affect research, practice or policy**

- CD8+T cells play a dominant role in controlling the disease in the setting of SEMA3A+ tumours, which might be exploited therapeutically.
- A comprehensive investigation of the mechanisms enabling cancer cells to break through several microenvironmental constraints will help to achieve a better PDAC control.

## Introduction

Pancreatic ductal adenocarcinoma (PDAC) is a malignancy of the exocrine pancreas and the deadliest cancer worldwide.^1^ Most patients present with an unresectable disease at diagnosis that is treated with chemotherapy-based regimens.^2^ Overall, PDAC is poorly responsive to available treatments.^2^ Evidence from studies addressing recurrences of PDAC following radical surgery suggests that pancreatic cancer is a systemic disease at presentation.^3–5^ As it stands, understanding the mechanisms of tumour progression and dissemination in PDAC is vital to improve patients’ outcomes in the long term. At histopathological level, PDAC tissues feature a prominent stromal reaction, abundant cancer-associated fibroblasts (CAFs) and macrophages, with T cells typically excluded. Expression profile analyses have evidenced two main subtypes of PDAC cells.^6–9^ These alternative cell states are not permanently encoded but rather defined by the integration of cell intrinsic (eg, specific allelic statuses) and cell extrinsic (eg, microenvironmental cues) factors.^10 11^ Moreover, PDAC tissues often show the coexistence of both basal-like and classical cells within the same tumour,^7 12^ which can be partially explained by the existence of spatially confined subtumour microenvironments (TMEs).^13^ Of the two epithelial PDAC cell states, the basal-like/squamous subtype is characterised by the loss of pancreatic endodermal identity and shows a more aggressive biological behaviour.^6 9^ Accordingly, basal-like/squamous cells accumulate in the advanced stages of the disease.^7^ Molecular signatures indicative of a challenging microenvironment (eg, hypoxia, fibrosis) represent core gene programmes of this subtype.^6 7^ This aligns with the possibility of inducing the basal-like/squamous subtype *ex vivo* by integrating specific TME cues into the culture medium.^11^

Genetic and epigenetic dysregulation of the Axon guidance pathway have been consistently reported in PDAC.^14–16^ Recently, Krebs *et al* showed the enrichment of axon guidance-associated gene sets in basal-like as well as high-grade PDAC.^17^ Furthermore, neuronal-like progenitor cell states have been reported in undifferentiated tumours^18^ and are positively selected in post-treatment tumours.^12^ Most of the previous studies have focused on investigating the role of members of the Slit/Robo axis on the PDAC malignant traits as well as its cell identity.^17 19–22^ Semaphorins are the largest family of axon guidance cues, which were originally identified as chemorepellent proteins in the nervous system.^23 24^ SEMA3A is a class 3 semaphorin, that is, secreted, whose elevated tissue expression is a negative prognostic marker in PDAC.^14 15^ Nonetheless, the functional role of semaphorins in PDAC remains to be elucidated. Here, we investigated whether the semaphorins signalling pathway contributes to shaping aggressive PDAC phenotypes. Integrating bulk and single-cell RNA-sequencing data with in situ analysis of PDAC tissues, we demonstrated that *SEMA3A* expression is prominent in the stroma of PDAC and specifically enriched in the epithelial cells of the basal-like/squamous subtype. We found that both cell-intrinsic and cell-extrinsic factors promoting the basal-like/squamous subtype induce expression of SEMA3A in PDAC cells. Mechanistically, SEMA3A acts cell autonomously to promote mesenchymal-like traits, including anoikis resistance, through the activation of focal adhesion kinase (FAK). In vivo, SEMA3A promotes the intratumour infiltration of macrophages and reduces the density of T cells. Finally, the depletion of macrophages with a CSF1R monoclonal antibody improved gemcitabine antitumour activity, particularly for SEMA3A expressing tumours.

## Results

### The expression of class 3 semaphorins is associated with the basal-like/squamous phenotype of PDAC

**Figure 1:**
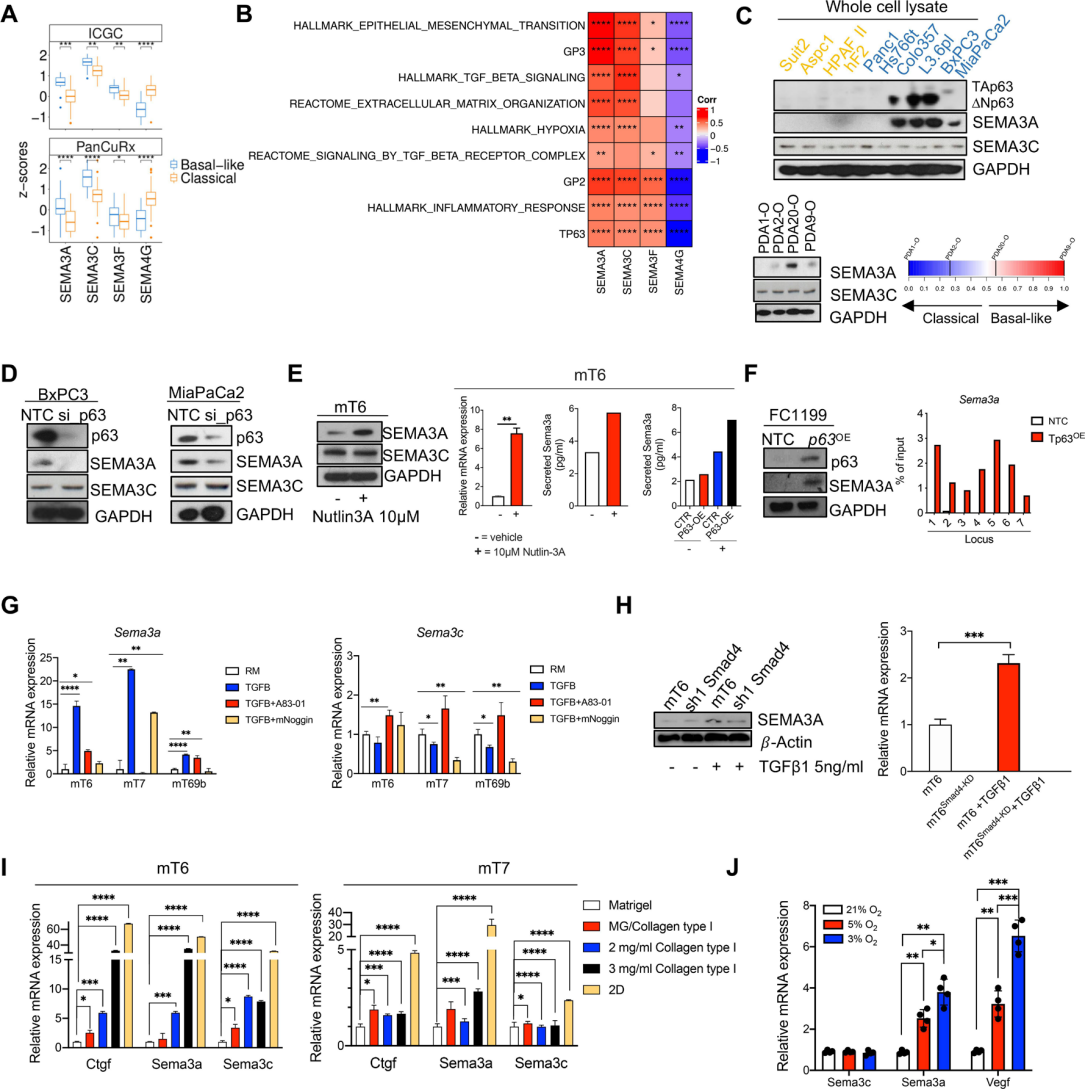
Cell intrinsic and cell extrinsic inputs eliciting SEMA3A expression in PDAC cells. (A) Boxplot of *SEMA3A*, *SEMA3C*, *SEMA3F* and *SEMA4G* Z-scores stratified by the Moffitt subtypes^9^ in the ICGC,^6^ and the PanCuRx^7^ cohorts. **p<0.01, ***p<0.001, ****p<0.0001 by Student’s t-test. (B) Heatmap showing correlation (Spearman’s correlation) between the indicated semaphorins and basal-like/squamous associated gene programmes in the ICGC cohort. GP2 and GP3 refers to the core gene programmes defining the squamous subtype in Bailey *et al*.^6^ All annotated boxes, p<0.05. (C) Upper panel, immunoblot analysis of p63, SEMA3A and SEMA3C in whole cell lysates of different human pancreatic cancer cell lines. Lower panel, immunoblot analysis of SEMA3A and SEMA3C in whole cell lysates of primary tumour organoids ordered based on their basal-like identity (from left to right increasing basalness). GAPDH, loading control. (D) Immunoblot analysis of p63, SEMA3A and SEMA3C in whole cell lysates from BxPC3 (left) and MiaPaCa2 (right) squamous cell lines transfected with either non-targeting control (NTC) of siRNA targeting p63. GAPDH as loading control. (E) Left panel, immunoblot analysis of SEMA3A and SEMA3C in whole cell lysates from mT6 treated with vehicle or Nutlin-3A (see the ‘Methods’ section). GAPDH, loading control. Changes in the expression (qPCR) or secretion (ELISA) of SEMA3A were detected in mT6 following Nutlin-3A treatment (right panel). (F) Immunoblot analysis of p63 and SEMA3A in KPC 2D cell lines (FC1199) transduced with either an empty vector (NTC) or a p63 ORF. On the right, anti-p63 ChIP-qPCR analysis of seven different genomic regions upstream of the promoter of *Sema3a*. The ChIP-qPCR signal of each sample was normalised to its own input. (G) qPCR showing changes in the expression of *Sema3a* (left) and *Sema3c* (right) relative to the reduced media condition (RM, without A83-01 and mNoggin) in three different tumour organoid cultures treated as indicated. Data are mean of three technical replicates. ****p<0.0001, **p<0.01, *p<0.05 by unpaired Student’s t-test. (H) Immunoblot analysis of SEMA3A in whole cell lysates from SMAD4 proficient and deficient mT6 organoids that were treated with either vehicle or TGF-β1 for 48 hours. β-actin was used as loading control (left panel). qPCR analysis (right panel) of SEMA3a in mT6 organoids treated as indicated. ***p<0.001 by Student’s t-test. (I) Changes in the expression of *Ctgf*, *Sema3a* and *Sema3c* in mouse tumour organoids (n=2) grown on substrate of increasing rigidity for 48 hours. Data are represented as mean value±SD (n=3 technical replicates). *p<0.05, ***p<0.001, ****p<0.0001 by Student’s t-test. (J) Changes in the expression levels of *Sema3c, Sema3a* and *Vegf* in mouse tumour organoids cultivated under different O_2_ concentration for 24 hours. Results are shown as mean±SD of four independent experiments. ***p<0.001, **p<0.01, *p<0.05 by Student’s t-test. GAPDH, glyceraldehyde 3-phosphate dehydrogenase.

The interrogation of three distinct PDAC transcriptomic datasets^6 7 25^ revealed that the expression level of four semaphorins significantly discriminated basal-like from classical tumours in the ICGC^6^ and the PanCuRx^7^ cohorts (figure 1A, online supplemental figure S1A). *SEMA4G* levels were enriched in classical tumours while the expression of *SEMA3A*, *SEMA3C* and *SEMA3F* was significantly enriched in basal-like PDAC. Furthermore, *SEMA3A* and *SEMA3C* showed the highest correlation with basal-like/squamous transcriptional signatures, including those indicative of a challenging microenvironment (eg, hypoxia and fibrosis) and of epithelial-to-mesenchymal transition (EMT) (figure 1B, online supplemental figure S1B). Therefore, we decided to focus on SEMA3A and SEMA3C. We identified higher SEMA3A expression in whole cell lysates from cells with prominent squamous features including expression of TP63/ΔNp63 (Colo357, L3.6pl, BxPC3 and MiaPaCa2) (figure 1C). Similarly, SEMA3A expression was higher in patient-derived organoids (PDOs) that classify as basal-like/squamous (figure 1C and online supplemental figure S1C). SEMA3C showed a more promiscuous pattern of expression in human cell lines and PDOs (figure 1C). Transient downregulation of *p63* was sufficient to reduce SEMA3A but not SEMA3C expression in MiaPaCa2 and BxPC3 cell lines (figure 1D). To understand the modulation of SEMA3A and SEMA3C expression during PDAC progression, we examined their expression levels across mouse PDAC cells displaying different *Trp53* allelic statuses, derived from tissues at different stages of disease progression. Overall, the expression level of both *Sema3a* and *Sema3c* was variable among stage-matched organoid cultures (ie, PanIN, tumour and metastases) from the KC (*Kras* ^LSL-G12D/+^;*Pdx-1*-Cre) and KPC (*Kras* ^LSL-G12D/+^;*Trp53* ^LSL-R172H/+^;*Pdx-1*-Cre) mouse models^26 27^ (online supplemental figure S1D). However, we found a trend towards an increase of *Sema3a* expression in advanced-stage cultures, and a significant difference between mM (ie, metastatic) and mN (ie, normal pancreas) cultures (online supplemental figure S1D) also in terms of protein expression and secretion (online supplemental figure S1E).

10.1136/gutjnl-2023-329807.supp1 Supplementary data

Our data also suggest that the loss of heterozygosity (LOH) of *Trp53* licenses SEMA3A expression in mouse PDAC cells (figure 1E, online supplemental figure S1F–H). In mouse PDAC, the in vivo progression towards invasive tumours is almost invariably associated with loss of heterozygosity (LOH) of *Trp53*.^27–30^ In human PDAC, the biallelic inactivation of *P53* is significantly enriched in basal-like/squamous tumours.^6^ mT organoid cultures established from KPC mice, differently from mM organoids, contain cells that retain the wild-type copy of *Trp53*.^28 29^ To deplete *Trp53* wild-type cells, we treated mT6 with 10 µM of Nutlin-3A^31^ (online supplemental figure S1F). Loss of the wild-type copy of *Trp53* in Nutlin-3A treated mT6 was associated with increased transcriptional and protein expression of SEMA3A, while levels of SEMA3C were unaffected (figure 1E, online supplemental figure S1G). Moreover, only in tumour organoids displaying LOH of *Trp53*, we could observe a significant induction of *Sema3a* expression following forced expression of p63 (figure 1E). To corroborate our findings, we leveraged mouse PDAC cell lines (referred to as KP^sh^ 32) where the loss of *Trp53* is contingent on the doxycycline-induced expression of a shRNA targeting *Trp53*. The genetic inactivation of *Trp53* in these cell lines led to a significant upregulation of *Sema3a* expression (online supplemental figure S1H). In line with the human data, forced expression of *p63* in the KPC cell line FC1199, which displays *Trp53* biallelic inactivation (online supplemental figure S1I), increased *Sema3a* expression (figure 1F). This was associated with increased occupancy of *Sema3a* promoter by p63 (figure 1F, online supplemental figure S1J). Conversely, transient downregulation of mutant *Kras* in KPC cell lines did not lead to changes in *Sema3a* expression while reducing the levels of genes downstream of mutant KRAS signalling such as *Nq01* and *Sema3c* 33 34 (online supplemental figure S1K).

### Environmental cues induce the expression of SEMA3A in mouse PDAC cells

Next, we investigated whether microenvironmental pressures that can lead to the basal-like cell state affected SEMA3A/3C expression. Three different mT cultures (mT6, 7, 69) grown in the presence of recombinant TGF-β1 (see methods) invariably showed increased *Sema3a* expression, while a context-dependent effect was observed for *Sema3c* (figure 1G). The stimulatory effect of TGF-β1 on *Sema3a* expression could be blocked either pharmacologically (figure 1G) or by the genetic downregulation of SMAD4 (figure 1H, online supplemental figure S1L). Matrix rigidity also affected Semaphorins expression. The cultivation of two different mTs (mT6 and mT7) in matrices of increasing rigidity significantly induced the expression of the mechanosensitive gene *Ctgf* as well as of *Sema3a* and *Sema3c,* although to a different extent (figure 1I). Finally, lowering the concentration of O_2_ significantly induced a dose-dependent expression of the hypoxia-responsive gene *Vegf*, of *Sema3a,* but not of *Sema3c* in FC1199 (figure 1J). Altogether, our results show that *Sema3a* is responsive to both cell intrinsic and cell extrinsic inputs that define aggressive PDAC phenotypes. These findings prompted us to investigate whether and how SEMA3A contributes to shape aggressive PDAC phenotypes.

### SEMA3A expression in normal and malignant pancreatic tissues

**Figure 2:**
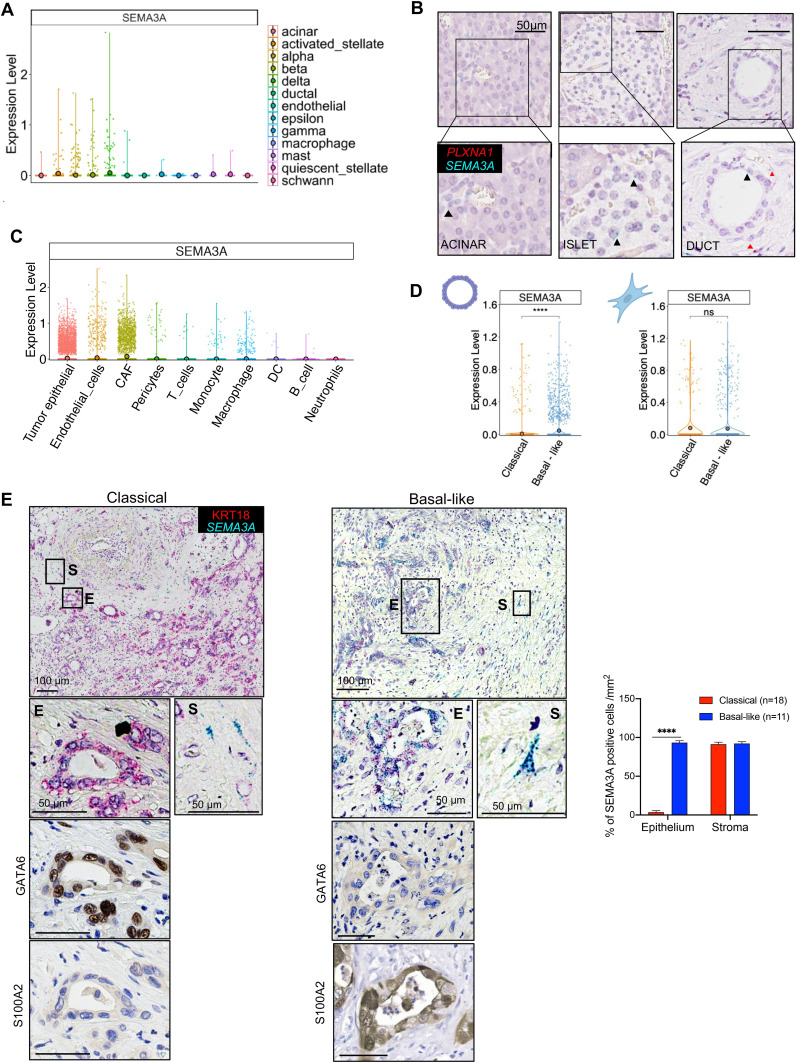
SEMA3A expression is selectively enriched in basal-like PDAC. (A) Violin plots of the normalised expression of *SEMA3A* in each annotated cell cluster from the integration of four different scRNA-Seq datasets^35–38^ of normal pancreatic tissues (see the ‘Methods’ section). (B) Representative ISH images showing rare *SEMA3A* (green) and *PLXNA1* (red) signals in acinar cells (left panel), islet cells (middle panel) and ductal cells (right panel). Scale bar, 50 µm. Insets show magnification of selected areas with visible signals for *SEMA3A* (black arrowheads) or *PLXNA1* (red arrowheads). (C) Violin plots of the normalised expression of *SEMA3A* in each annotated cell cluster from the integration of 4 different scRNA-Seq datasets^7 40–42^ of pancreatic cancer tissues (see the ‘Methods’ section). (D) Epithelial and fibroblasts expression of *SEMA3A* in individual cells from PDAC cases almost exclusively composed by either classical or basal-like cells (see online supplemental figure S2H). ****p<0.0001 and ns, not significant by Wilcoxon and Mann-Whitney. (E) Left panel, representative ISH images showing expression of *SEMA3A* in the epithelial (CK18+) and stromal (CK18−) compartment of a pancreatic cancer tissue subdomain classified as classical based on expression of GATA6. Right panel, representative ISH images showing expression of *SEMA3A* in the epithelial and stromal compartment of a tumour area classified as basal-like based on the expression of S100A2 and lack of GATA6 expression. Scale bars as indicated. Quantification is provided as percentage of positive cells (see also online supplemental figure S3B) in the selected area. ****p<0.0001 by unpaired Student’s t-test. ISH, in situ hybridisation.

Elevated tissue expression of SEMA3A has been previously linked with dismal outcomes in PDAC.^14^ Here, we sought to clarify the major cellular sources of SEMA3A in pancreatic tissues. Integration of available scRNA-Seq data^35–38^ with in situ hybridisation (ISH) analysis of normal pancreatic tissues revealed that rare epithelial cells (mostly neuroendocrine cells) express low levels of *SEMA3A* and its receptor *PLXNA1* (figure 2A,B). Accordingly, the analysis of bulk transcriptomic data revealed that levels of *SEMA3A* were significantly higher in tumour versus normal pancreatic tissues^39^ (online supplemental figure S2A). To specifically link epithelial SEMA3A expression to molecular features of aggressive PDAC, we leveraged the transcriptomic data of the PanCuRx cohort which were generated following laser-capture microdissection of the epithelial compartment.^7^ Samples were stratified based on the *SEMA3A* expression status (either high or low, see the ‘Methods’ section). *SEMA3A* ^high^ tumours were enriched for basal-like subtypes (online supplemental figure S2B) and major imbalances of the mutant *KRAS* allele (online supplemental figure S2C).

10.1136/gutjnl-2023-329807.supp2 Supplementary data

10.1136/gutjnl-2023-329807.supp3 Supplementary data

To further corroborate the link between *SEMA3A* expression in epithelial cells with the basal-like transcriptional cell state, we interrogated scRNA-Seq data of human PDAC tissues.^7 40–42^ Following harmonisation of the four datasets,^43^ cell type annotation was performed using singleR^44^ and the Human Primary Cell Atlas^44^ (online supplemental figure S2D). As expected, epithelial and stromal cells represented the most populated cell clusters. Next, we inferred copy-number alterations^45^ in the ductal cell clusters to identify malignant cells and exclude normal epithelial cells (online supplemental figure S2E). CAFs were annotated in the stromal cell clusters by post hoc analysis using known gene signatures^46^ (online supplemental figure S2F). *SEMA3A* expression was not restricted to epithelial cells but rather prominent in stromal elements (figure 2C). Expression of SEMA3A receptor (*PLXNA1*) and coreceptor (*NRP1*) was rather promiscuous in PDAC tissues, which suggests that many cell types might be responsive to this axon guidance cue (online supplemental figure S2G). When considering cases with the highest proportion of basal-like and classical cells across the four datasets (online supplemental figure S2H), significant differences in terms of *SEMA3A* expression were restricted to the malignant epithelium (figure 2D). In scRNA-seq data from an autochthonous mouse model of PDAC,^47^ *Sema3a* expression was higher in the epithelial compartment and particularly enriched in basal-like cells (online supplemental figure S2I). Finally, we performed ISH for *SEMA3A* on human PDAC tissues (n=29) and classified neoplastic cells as either classical or basal-like/squamous based on the expression of markers of the two subtypes (online supplemental figure S3A). As expected, PDAC tissues displayed marked intratumour heterogeneity with coexistence of basal-like and classical neoplastic cells (online supplemental figure S3A). For each tumour tissue, we identified 1 mm^2^ area exclusively occupied by either classical or basal-like/squamous cells and evaluated *SEMA3A* in the epithelial and stromal compartments. In keeping with the scRNA-Seq data, *SEMA3A* was almost exclusively detected in basal-like epithelial cells while detectable in stromal elements surrounding both classical and basal-like cells (figure 2E and online supplemental figure S3B). In sum, our analysis shows that *SEMA3A* expression is not restricted to epithelial cells in PDAC tissues, yet it is mostly confined to basal-like/squamous epithelial cells. Therefore, we sought to investigate the role of tumour cells derived SEMA3A in pancreatic progression.

### SEMA3A activates the PI3K/Akt signalling pathway in mouse PDAC cells

**Figure 3:**
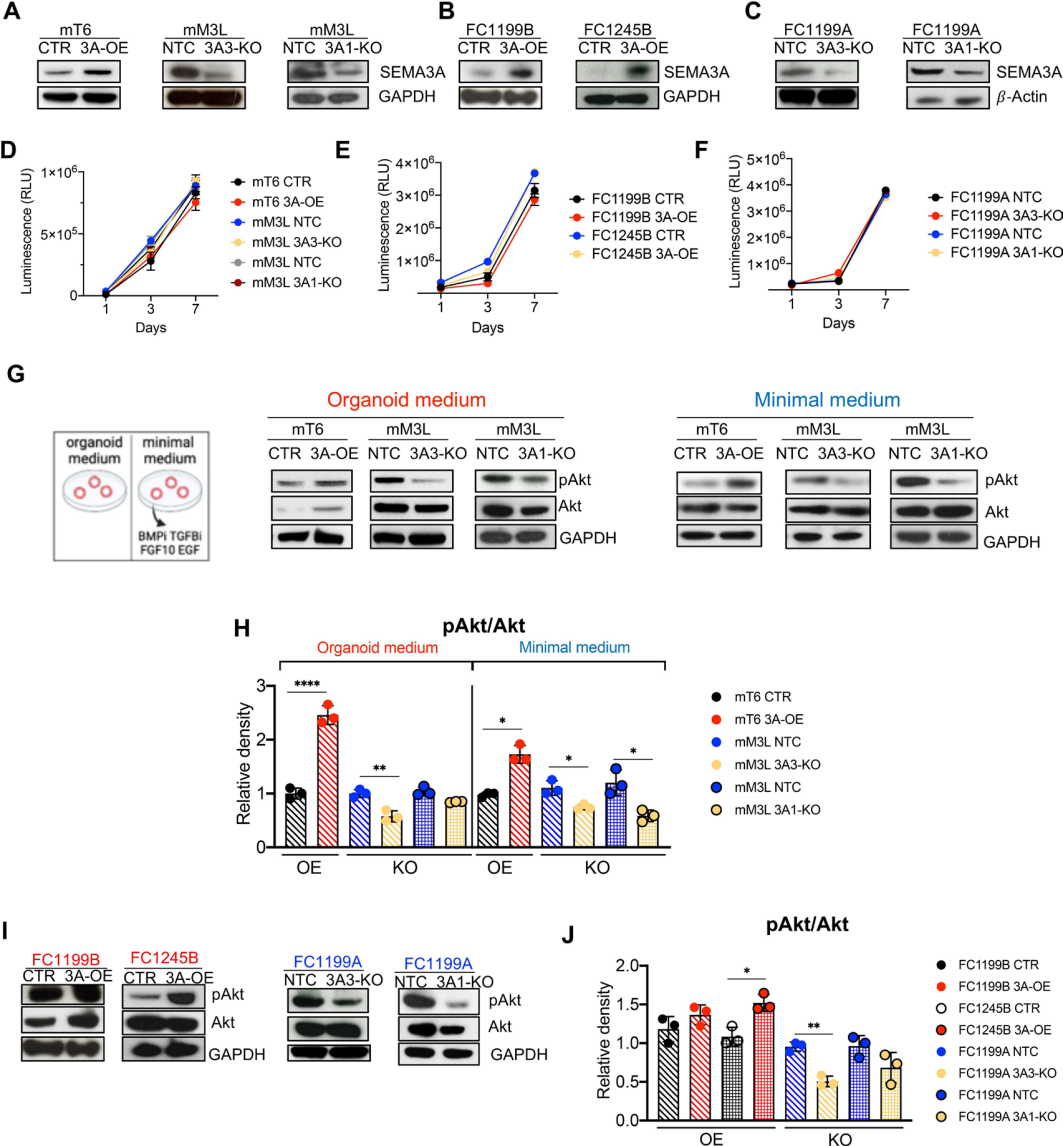
SEMA3A promotes the activation of PI3K/Akt in mouse pancreatic ductal adenocarcinoma (PDAC) cells. (A–C) Immunoblot analyses of SEMA3A in whole cell lysates from mouse tumour (mT6) organoids, mouse metastatic (mM3L) organoids and KPC 2D cell lines (FC1199 and FC1245) following either overexpression (OE) or genetic knockout (KO); GAPDH was used as loading control in A and B while β-actin was used in C. (D–F) Proliferation (as total luminescence) measured over the course of 7 days of either cell lines or organoid cultures from A to C. The suffix L for mM3 denotes that the culture was established from a liver metastasis. The suffixes A and B for FC1199 and FC1245 denote clonal populations displaying high and low levels of SEMA3A, respectively. NTC or CTR denotes cultures stably transduced with a mock control. (G) Immunoblot analyses of the indicated proteins in whole cell lysates from mouse organoid cultures (mT6 and mM3L) cultivated in either organoid medium or in minimal medium. GAPDH was used as loading control. (H) Bar plots showing the quantification of changes in the phosphorylated levels of p-AKT as relative density of the total protein level. Data are presented as means±SD of three biological replicates. ****p<0.0001, **p<0.01, *p<0.05 by Student’s t-test corrected for multiple comparison using the Holm-Sidak method. (I) Immunoblot analyses of the indicated proteins in whole cell lysates from mouse pancreatic cancer cell lines following either overexpression (OE) or genetic knockdown (KO) of SEMA3A. GAPDH was used as loading control. (J) Bar plots showing the quantification of changes in the phosphorylated levels of p-AKT as relative density of the total protein level. Data re presented as means±SD of three biological replicates. **p<0.01, *p<0.05 by Student’s t-test corrected for multiple comparison using the Holm-Sidak method. GAPDH, glyceraldehyde 3-phosphate dehydrogenase.

To understand whether and how dysregulated SEMA3A levels contribute to promote malignancy of PDAC cells, we performed genetic perturbation experiments using both mouse PDAC cell lines and organoids. KPC-derived cell lines (FC1199, FC1245 and FC1242) display mesenchymal-like features (online supplemental figure S4A) and high levels of *Sema3a*. Therefore, we derived subclones displaying a reduced expression of the gene (online supplemental figure S4B). *Sema3a* ^low^ FC1199 and FC1245 subclones (designated by the B suffix) and mT6 organoids were stably transduced with a vector carrying an open-reading frame for *Sema3a* (figure 3A,B and online supplemental figure S4C,D). Cas9-expressing mM3L organoids and *Sema3a^high^* FC1199 and FC1245 monolayer cell cultures (designated by the A suffix) were transduced with two different gRNAs targeting *Sema3a* (figure 3A,C and online supplemental figure S4C,E). Genetic manipulation of *Sema3a* also resulted in coherent changes in the level of the secreted proteins in the cultures conditioned media (online supplemental figure S4F). No difference in cell viability over the course of 7 days was observed for mouse organoids (figure 3D) as well as for 2D cultures (figure 3E,F) displaying different levels of *Sema3a*.

10.1136/gutjnl-2023-329807.supp5 Supplementary data

Next, we evaluated whether the dysregulation of *Sema3a* in mouse PDAC cells affected fluxes through the major signalling pathways, that is, MAPK and PI3K/Akt pathways. To test the effect of the culturing medium on pathways' modulation, we cultured organoids (mT6 and mM3L) in standard and minimal media (depleted of growth factors and TGF-β inhibitors). Regardless of the culturing media, SEMA3A promoted activation of the PI3K/Akt pathway in organoids (figure 3G,H). Similar changes were observed in monolayer cell cultures following S*ema3a* perturbation (figure 3I). Conversely, the effect of *Sema3a* dysregulation on the activation of the MAPK pathway was variable across cultures and culture conditions (online supplemental figure S4G–J). Overall, our data suggest that SEMA3A promotes PI3K/Akt activation in mouse PDAC cultures independently of the culture environment and matrix dimensionality.

### SEMA3A promotes increased migration, anoikis resistance and increases lung metastases

Expression of SEMA3A in human PDAC tissue correlates with EMT gene programmes (figure 1B), and transcription of *Sema3a* in mouse cultures is induced by TGF-β1 (figure 1H). In organoid cultures, the modulation of *Sema3a* was associated with significant changes in mesenchymal (Vimentin) or epithelial (E-cadherin) markers only in a minimal medium (online supplemental figure S5A,B). Furthermore, neither the knockout nor the overexpression of *Sema3a* significantly influenced TGF-β1 induction of EMT transcription factors expression in organoids (online supplemental figure S5C,D). As expected, TGF-β1 failed to induce *Sema3a* transcription in knockout cells (online supplemental figure S5D). In monolayer cell cultures (FC1199 and FC1245), modulation of *Sema3a* had no significant effect on the expression of EMT markers (online supplemental figure S5E,F).

10.1136/gutjnl-2023-329807.supp6 Supplementary data

**Figure 4:**
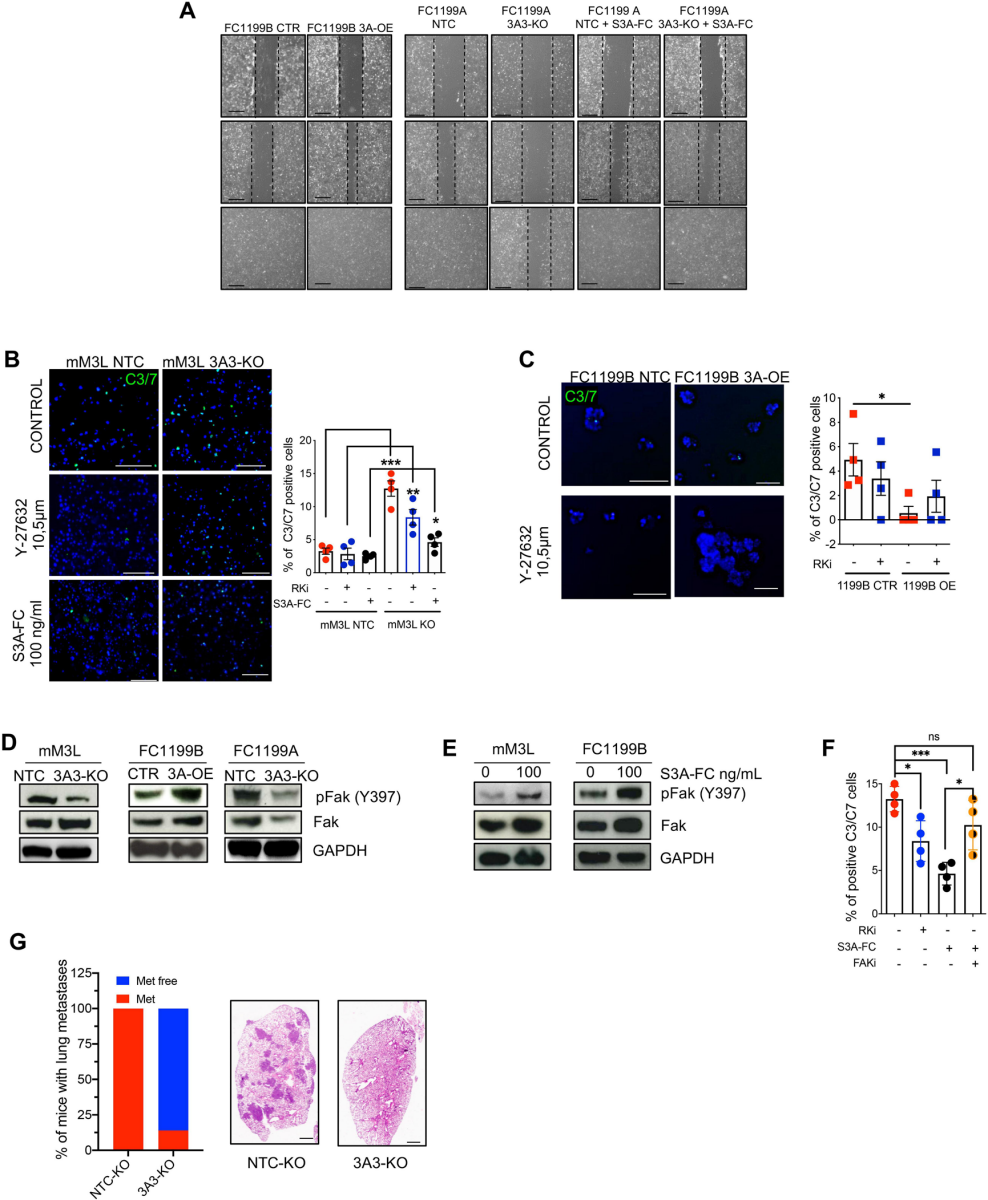
SEMA3A promotes anoikis resistance and increases lung metastases. (A) Representative photographs of the wound area taken immediately after (0), 8 and 24 hours after the incision for FC1199A and B cell lines stably transduced with either non-targeting or control vectors (NTC, CTR), SEMA3A ORF (OE) or gRNAs targeting SEMA3A (KO). FC1199A NTC and KO cells were also treated with recombinant SEMA3A. The experiment was performed in quadruplicate. (B) Representative immunofluorescence images of the anoikis assay (see methods) performed on poly-HEMA coated plate for mM3L NTC and KO treated vehicle (Control), with a RhoK inhibitor (Y-27632), or with recombinant SEMA3A (S3A-FC). Scale bars, 100 µm. Quantification of four independent experiments is provided in the box plot on the right. ***p<0.001, **p<0.01, *p<0.05 by Student’s t-test. (C) Representative immunofluorescence images of the anoikis assay (see the ‘Methods’ section) performed for FC1199B CTR and OE treated with vehicle (Control) or a RhoKi. Scale bars, 100 µm. Quantification of four independent experiments is provided in the box plot on the right. *p<0.05 by Student’s t-test. (D) Immunoblot analysis of phospho-FAK, and total FAK in whole cell lysates from mM cultures and KPC cell lines with different SEMA3A genotypes (eg, KO or OE). (E) Immunoblot analysis of phospho-FAK and total FAK in whole cell lysates from mM3L and FC1199B treated with recombinant SEMA3A. GAPDH was used as loading control in D and E. (F) Quantification of apoptotic cells from the anokis assay of the SEMA3A knockout mM3L treated with vehicle, with the RhoK inhibitor (RKi), the recombinant SEMA3A (S3A-FC) or the combination of S3A-FC and defactinib (FAKi). Data are displayed as mean ± SD of four technical replicates. *p<0.05, ***p<0.001 by Student’s t-test. (G) Stacked bar plot displaying the percentage of mice (n=7 per group) displaying lung metastases on tail-vein injection of *Sema3a* proficient (NTC) and deficient (KO) cells. Scale bar 1 mm. GAPDH, glyceraldehyde 3-phosphate dehydrogenase; Poly-HEMA, poly(2-hydroxyethyl methacrylate).

Next, we asked whether SEMA3A functionally contributes to EMT traits in mouse PDAC cells. The manipulation of *Sema3a* expression in mouse PDAC cultures had a significant effect on their migratory capability (figure 4A). The wound healing assay showed that *Sema3a* significantly promoted the migration of FC1199B cells (figure 4A). In keeping with that, the treatment with recombinant SEMA3A rescued the effect of gene knockout on the migratory capacity of FC1199A cells (figure 4A, right panel). Next, we set up an anoikis assay for both monolayer cell cultures and organoids (see methods). The depletion of *Sema3a* from mM organoids significantly increased apoptotic cell death (figure 4B). Similar to the anoikis inhibitor Y27632 (Rho-associated kinase inhibitor, RhoKi), exogenous supplementation of recombinant SEMA3A significantly reduced apoptotic cell death. In keeping with that, overexpression of *Sema3a* in FC1199 significantly reduced cell death of cells grown in suspension (figure 4C). To test whether the resistance to anoikis was mediated by the canonical SEMA3A-NRP1 axis, we measured the anti-anoikis effect of SEMA3A following silencing of either *Nrp1* or the main coreceptor *Plxna1* (online supplemental figure S5G). Silencing of *Nrp1,* and to a lesser extent of *Plxna1,* significantly prevented the protective effect of SEMA3A against anoikis (online supplemental figure S5G). FAK is an important regulator of cell survival with an established role in mediating anoikis resistance.^48^ We found that cancer-cell-derived SEMA3A induces activation of FAK (autophosphorylation at Tyr 397) (figure 4D), which was instead reduced on silencing of SEMA3A receptors (online supplemental figure S5H). Of note, silencing of SEMA3A receptors, particularly of PLXNA1, reduced fluxes through main signalling pathways (online supplemental figure S5H). Treatment of mouse PDAC cultures with recombinant SEMA3A stimulated FAK autophosphorylation (figure 4E) and FAK inhibition with the selective inhibitor defactinib (FAKi) counteracted the SEMA3A protective effect against anoikis (figure 4F and online supplemental figure S5I). Anoikis resistance is a hallmark of metastatic cells.^49^ Therefore, we injected both *Sema3a* proficient and deficient cells directly into the circulation to model postintravasation steps of the metastatic process, including survival into the circulation. In accordance with the in vitro experiments, *Sema3a* proficient cells rapidly colonised the lung parenchyma as opposed to *Sema3a* deficient cells (figure 4G). Overall, our data suggest that *Sema3a* is dispensable for the induction of an EMT transcriptional phenotype driven by microenvironmental cue (eg, TGF-β); however, it is an important mediator of mesenchymal-like traits in PDAC.

### SEMA3A expression sustains basal-like/squamous gene programmes in PDAC

**Figure 5:**
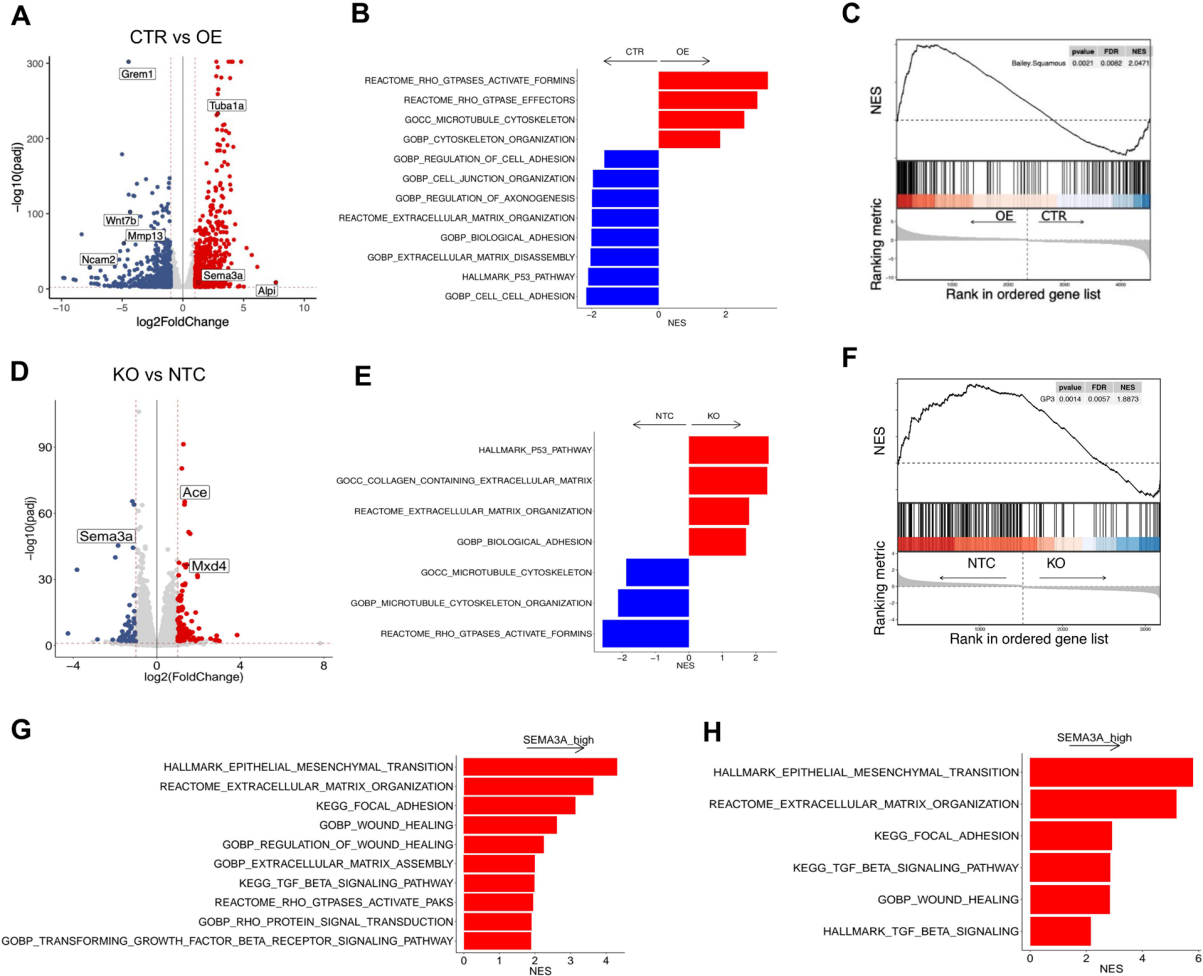
Transcriptomic changes following SEMA3A perturbation. (A) Volcano plot of the differences in gene expression between control (CTR, n=3) and *Sema3a* overexpression (OE n=3). Indicated are some of the genes with log2FC expression≥2 and adjusted p<0.05. See online supplemental table S1 for the full list of differentially expressed genes. (B) Enrichment of selected pathways (GSEA) when comparing FC1199B *Sema3a^low^* (CTR) and FC1199B overexpressing *Sema3a* (OE). See also online supplemental table S2. (C) GSEA plot evaluating the Squamous signature^6^ on *Sema3a* overexpression (OE) in FC1199B cells. (D) Volcano plot of the differences in gene expression between control (NTC, n=3) and *Sema3a* knockout (KO, n=3). Indicated are some of the genes with log2FC expression≥2 and adjusted p<0.05. See online supplemental table S3 for the full list of differentially expressed genes. (E) Enrichment of selected pathways (GSEA) when comparing *Sema3a* proficient (NTC) and deficient (KO) FC1199A cells. See also online supplemental table S4. (F) GSEA plots evaluating the Gene Programme 3 (GP3, TGFβ pathway)^6^ on *Sema3a* knockout in FC1199A cells. (G–H) Enrichment of selected pathways when comparing SEMA3A high and low tissues from the ICGC^6^ (G) and the PanCuRx^7^ (H) cohorts. See also online supplemental tables S5–S8. In B, E, G and H, GSEA was performed using gene sets from Hallmark, GO, KEGG, Reactome and HP databases in MsigDB library. Displayed gene sets that passed false discovery rate <0.05. GSEA, gene set enrichment analysis.

To identify pathways downstream of *Sema3a* involved in promoting PDAC aggressiveness, we performed transcriptomic analysis on mouse PDAC cells of different genotypes. The overexpression of *Sema3a* had significant effects on the transcriptome of FC1199B with over 2000 genes significantly upregulated and downregulated (figure 5A, online supplemental table S1). The genetic knockout of *Sema3a* led to a similar degree of transcriptomic changes (figure 5D, online supplemental table S3). As shown in figure 5A, the forced overexpression of *Sema3a* was associated with the significant downregulation of *Grem1*, a BMP inhibitor that has been shown to promote epithelialisation of mesenchymal PDAC cells.^50^ Next, we performed gene set enrichment analysis on the list of differentially expressed genes using the GSEA method^51^ (figure 5B,E). Following the overexpression of *Sema3a*, we observed the enrichment of gene programmes related to cytoskeleton remodelling and the activity of Rho GTPases (figure 5B, online supplemental table S2). In keeping with that, the knockout of *Sema3a* led to the reduced representation of the same gene programmes (figure 5E, online supplemental table S4). Secreted SEMA3A generally induces growth cone collapse in neurons by acting as either chemorepellent or chemoattractant through microtubule and actin reorganisation.^52^ Moreover, SEMA3A is reported to interact directly or indirectly with multiple GTPases, including Rho GTPases.^52^ Of note, overexpression of *Sema3a* was also associated with a reduced representation of axon guidance gene sets, which we linked to the reduced expression of Slit/Robo genes. When looking at signatures of aggressive human PDAC, we found that SEMA3A^high^ cells presented higher ‘squamousness’ than SEMA3A^low^ cells. Furthermore, SEMA3A deficient cell lines showed a significant reduction of the GP3 gene programmes defined by Bailey *et al* 6 based on inferred activity of the TGF-β pathway (figure 5C,F). Moreover, gene programmes related to EMT, the TGF-β pathway, the activation of FAK, Rho GTPases and wound healing were significantly enriched in *SEMA3A^hig^* ^h^ tumours both in the ICGC^6^ and the PanCuRx^7^ cohorts (figure 5G,H, online supplemental tables S5–S8).

10.1136/gutjnl-2023-329807.supp4 Supplementary data

### SEMA3A promotes PDAC progression in vivo

**Figure 6:**
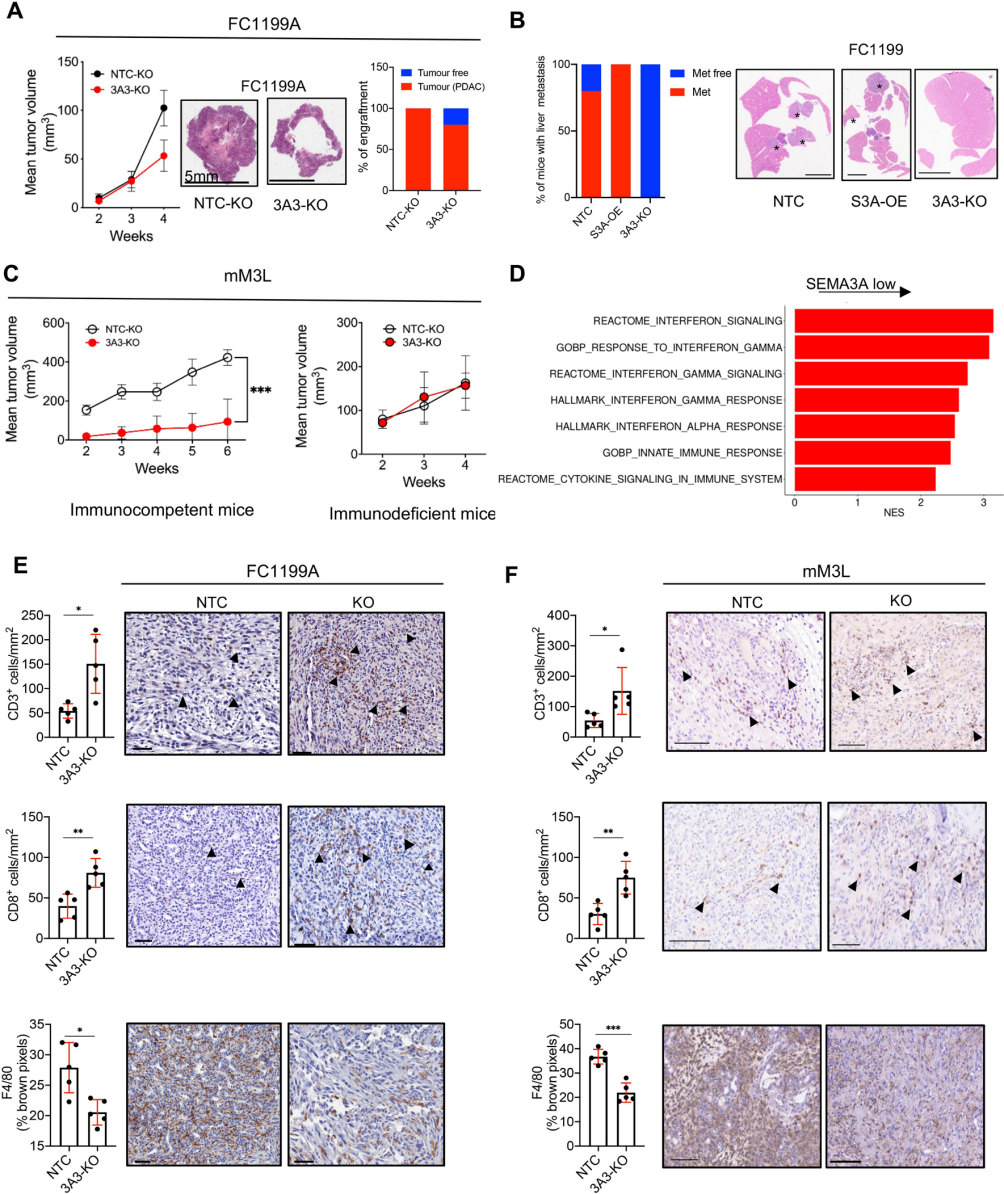
SEMA3A promotes growth of PDAC cells through modification of the tumour microenvironment. (A) On the left, line graph showing tumour volumes (mm^3^) of pancreatic masses detected on the orthotopic injection of 1×10^5^ cells from FC1199A (n=11/group). Means±SD are shown; difference not reaching statistical significance by Student’s t-test. Middle panel, histological images of transplanted tumours; scale bar as indicated. On the right, stacked bar plot displaying the percentage of tumour bearing mice in the two cohorts (NTC and KO). (B) Stacked bar plot displaying the percentage of mice (n=5 per group) displaying liver metastases on intrasplenic injection of *Sema3a* proficient (NTC) deficient (KO) and overexpressing (OE) FC1199 cells. Scale bar 1 mm. (C) Line graph showing tumour volumes (mm^3^) of pancreatic masses detected on injection of 1×10^6^ cells from mM3L organoids into the pancreata of immunocompetent (n=10 mice per group, left panel) or immunodeficient (n=5 mice per group, right panel) mice. Means±SD are shown. ****p<0.001 by two-way ANOVA with Sidak’s test for multiple comparison. Tumour volume was assessed using Vevo 2100 System with a MS250, 13–24 MHz scanhead (Visual Sonics). (D) Enrichment of selected pathways when comparing tissues from *Sema3a* deficient (n=3) and proficient (n=3) tumours. GSEA was performed using gene sets from Hallmark, GO, Reactome and HP databases in MsigDB library. Displayed gene sets that passed false discovery rate <0.05. See online supplemental tables S9 and S10 for details. (E–F) Representative immunohistochemical staining for T cells markers (CD3 and CD8) and the macrophage marker F4/80 in pancreatic tissues from mice transplanted with: (E) FC1199A cells or (F) mM3L organoid cultures stably transduced with either non-targeting vector (NTC) or gRNA targeting *Sema3a* (KO). Scale bars, 50 µm. Quantification is provided on the left as mean±SD (see the ‘Methods’ section). At least five individual areas per case and a minimum of five mice/arm were evaluated. Arrowheads indicate positive staining. ANOVA, analysis of variance.

Next, we sought to assess the in vivo phenotypic consequences of *Sema3a* dysregulation. First, we evaluated whether and how SEMA3A influenced tumour growth pattern and kinetics. Overall, *Sema3a* expressing cell lines (FC1199A and FC1245) generated larger tumours with a solid growth pattern as opposed to the cystic pattern observed for *Sema3a* deficient or low cells (figure 6A, online supplemental figure S6A,B). Moreover, 2 out of 11 mice transplanted with *Sema3a* deficient FC1199 cells did not show any detectable mass while tumour masses invariably developed from *Sema3a* expressing cells (figure 6A). Furthermore, SEMA3A expressing tumours (from FC1199 cells) colonised the liver parenchymal more efficiently than the knockout cells after intrasplenic injection (figure 6B).

10.1136/gutjnl-2023-329807.supp7 Supplementary data

Next, we generated grafts based on the transplantation of mouse organoids. Immunocompetent mice transplanted with syngeneic organoid cultures display delayed kinetics of in vivo tumour progression as opposed to PDAC established from monolayer cell cultures.^28 29 53^ Nonetheless, this model system permits a better evaluation of the effect of genetic perturbation on tumour progression in vivo.^28 54^ *Sema3a* deficient mM3L organoids generated smaller tumours in an immunocompetent host (figure 6C and online supplemental figure S6C) while no difference in tumour growth kinetics was observed in immunodeficient hosts (figure 6C). These results suggest the involvement of the immunity in mediating the in vivo protumourigenic effects of SEMA3A in this model system. RNA-Seq analysis of tumour tissues collected at endpoint from mice transplanted with 2D cell lines did not reveal striking transcriptomic changes between the two groups (online supplemental figure S6,D, online supplemental table S9). However, gene-set enrichment analysis showed the overrepresentation of terms related to inflammation and interferon-related pathways in tumours lacking SEMA3A (figure 5D, online supplemental table S10). The characterisation of the immune microenvironment in murine pancreatic tumours established from cells displaying different *Sema3a* statuses suggested profound remodelling of the TME by tumour-derived SEMA3A (figure 6E,F and online supplemental figure S6E,F). Immunophenotyping showed that SEMA3A promoted abundant intratumoural infiltration of macrophages (F4/80+cells) while concurrently reducing intratumoural density of T cells (CD3+and CD8+ T cells) (figure 6E,F, online supplemental figure S6E). The in situ immunophenotypic results were confirmed by FACS analysis of whole-tumour tissues (online supplemental figure S6F), which further showed no significant SEMA3A-induced changes in other myeloid or T cell (eg, Treg) compartments. Finally, we analysed the transcriptomic data from the ICGC cohort dividing tissues based on the expression of *SEMA3A* (see the ‘Methods’ section). Consistent with our findings in the mouse models, transcriptional signatures of tumour-associated macrophages (TAMs) were significantly enriched in *SEMA3A* high tumours (online supplemental figure S6G). These results prompted us to investigate the role of tumour-derived SEMA3A on macrophage recruitment and polarisation.

### Increased intratumoural infiltration of TAMs contributes to the aggressive behaviour of SEMA3A high tumours

**Figure 7:**
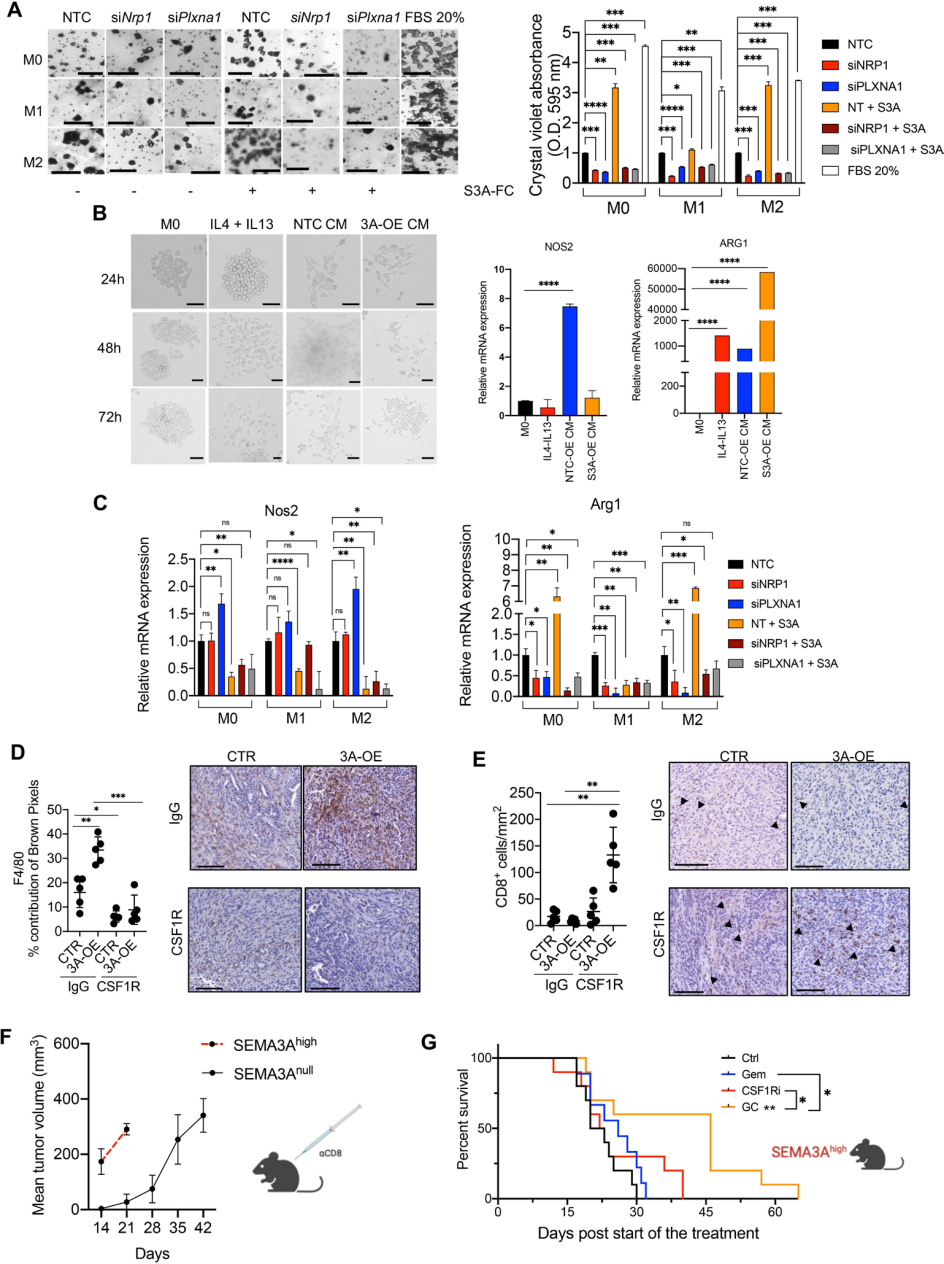
Increased intratumoural infiltration of TAMs contributes to the aggressive behaviour of SEMA3A high tumours. (A) Bright-field images of migrated macrophages in the transwell assay (see methods, left panel). The quantification is provided on the right as bar plots displaying mean±SD of the optical density values from three technical replicates. ***p<0.001, **p<0.01, *p<0.05 by unpaired Student’s t-test. (B) Left panel, brightfield images of mouse RAW 264.7 cells treated as indicated for up to 72 hours. From left to right, untreated (M0), combination of IL4+IL13, conditioned media from control cells and conditioned media from cells overexpressing SEMA3A. Right panel, qPCR showing relative mRNA expression of *Nos2* and *Arg1*. Data are mean of three technical replicates. ****p<0.0001 by Student’s t-test. (C) qPCR showing relative mRNA expression of *Nos2* (left) and *Arg1* (right). Data are mean of three technical replicates. ***p<0.001, **p<0.01, *p<0.05 by unpaired Student’s t-test. (D) Representative immunohistochemical staining for the macrophage marker F4/80 in pancreatic tissues from mice transplanted with FC1199B cells stably transduced with either mock (CTR) or a vector carrying *Sema3a* ORF (3 A-OE) treated with control IgG or CSF1R monoclonal antibody. (E) Representative immunohistochemical staining for the cytotoxic T cell marker (CD8) in pancreatic tissues from mice transplanted with FC1199B cells stably transduced with either mock (CTR) or a vector carrying *Sema3a* ORF (3 A-OE) treated with control IgG or CSF1R monoclonal antibody. In E and F, scale bars, 50 µm. Quantification is provided on the left as mean±SD (see the ‘Methods’ section). At least five individual areas per case and a minimum of five mice/arm were evaluated. Arrowheads indicate positive staining. (F) Line graph showing tumour volumes (mm^3^) of pancreatic masses detected in mice transplanted with SEMA3A overexpressing (SEMA3Ahigh) or null (SEMA3Anull) cells treated with αCD8 (CD8, n=10). (G) Kaplan-Meier survival analysis of mice transplanted with SEMA3A high cells and treated with control IgG (Ctrl, n=10), Gemcitabine (Gem, n=10), αCSF1R (CSF1Ri, n=10) or combination of Gemcitabine and αCSF1R (GC, n=10). Statistical differences identified by log-rank test.

To model the potential effect of SEMA3A on macrophages’ recruitment, we used the transwell migration assay. The monocyte/macrophage cell line RAW 264.7 was first polarised towards M1-like or M2-like macrophages (online supplemental figure S7A, see the ‘Methods’ section). Then, polarised and non-polarised macrophages were seeded with Matrigel in transwell to perform an invasion assay. As expected, a medium containing 20% FBS supported the invasion in all macrophages’ phenotypes (figure 7A). Similarly, recombinant SEMA3A promoted the invasion of all macrophages’ phenotypes, although to a different extent (figure 7A). Silencing of the receptors (either *Nrp1* or *Plxna1*) completely abrogated the chemoattractive effects of SEMA3A (figure 7A).

10.1136/gutjnl-2023-329807.supp8 Supplementary data

Next, we evaluated the effect of SEMA3A on the polarisation of macrophages using both RAW 264.7 and bone-marrow-derived monocytes. We grew RAW 264.7 in standard medium, in medium containing a cocktail of cytokine inducing the M2-like state (IL4 and IL13), and in conditioned media from SEMA3A proficient and deficient cells. As expected, the combined treatment with IL4 and IL13 induced morphological and molecular activation (figure 7B) of the macrophages, with increased expression of *Arg1* (marker of M2-like macrophages) and a slight (although not significant) reduction of the expression of the M1-like gene *Nos2*. As opposed to the conditioned medium from *Sema3a* deficient cells, the conditioned medium from SEMA3A expressing tumour cells significantly induced *Arg1* expression without eliciting *Nos2* expression. Coherently, the treatment of bone-marrow-derived monocytes with recombinant SEMA3A induced protein and mRNA expression of M2-like markers (online supplemental figure S7B,C). We then evaluated the effect of receptor knockdown on the polarising effect of SEMA3A. In unperturbed conditions, SEMA3A treatment reduced *Nos2* expression in all macrophages’ subsets, while inducing *Arg1* expression in M0, M2 but not M1 (figure 7C). Following silencing of the receptors, SEMA3A induced changes in *Nos2* and *Arg1* expression were prevented in all subsets (figure 7C). The in vitro data were consistent with the higher density of CD206+macrophages in tumour tissues from SEMA3A expressing cells as evidenced by both FACS analysis (online supplemental figure S6F) and immunophenotyping (online supplemental figure S7D)

To understand whether the reduced T cells infiltration of SEMA3A^high^ tumours was due, at least in part, to the abundance of TAM at the tumour bed, we targeted macrophages using a monoclonal antibody against CSF1R (αCSF1R). As shown in online supplemental figure S7E, immunocompetent mice were treated daily with αCSF1R 3 days prior the transplantation with SEMA3A proficient and deficient cells along with the control. The treatment with αCSF1R continued every other day until endpoint and tumour growth monitored by manual palpation and ultrasound imaging. At endpoint, we observed a significant reduction of intratumoural infiltration by macrophages (F4/80+cells) in tumours from mice treated with αCSF1R regardless of the SEMA3A status (figure 7D). Cytometric analyses of blood samples from tumour-bearing mice also confirmed the reduction of F4/80+cells with no significant effect on Ly6C^+^Ly6G^+^ or Ly6C^+^ cells (online supplemental figure S7F), which is in line with the inhibition of CSF1R in mouse PDAC using a small molecule.^10^ Only in tumours established by SEMA3A overexpressing cells, the depletion of macrophages was associated with increased intratumoural infiltration by CD8+T cells (figure 7E).

Given the prominent difference in T cell infiltration following macrophages depletion, we sought to explore whether CD8+T cell depletion would have a different effect on the growth of SEMA3A^high^ and SEMA3A^low^ tumours. The depletion of CD8+T cells led to the rapid progression of the disease of SEMA3A^high^ so that mice succumbed to the disease within 7 days from the beginning of the treatment (figure 7F). This result suggested that CD8+T cells play a dominant role in controlling the disease in the setting of SEMA3A+tumours. Next, we tried to assess whether depletion of macrophages had differential effect on the disease control achievable through pharmacological treatment. First, we tested the effect of CSF1R inhibition alone or in combination with gemcitabine on the survival of mice bearing tumours from either *Sema3a* high or low cells (online supplemental figure S7G). In line with their less aggressive behaviour, SEMA3A low tumours responded to all the treatments, yet gemcitabine monotherapy did not reach statistical significance (p=0.08) (online supplemental figure S7H). SEMA3A high tumours responded poorly to both gemcitabine and CSF1R inhibition as monotherapy, and only the combination significantly extended the survival of the mice (figure 7G). On depletion of CD8+T cells, the combination lost its antitumour activity in *Sema3a* expressing tumours (online supplemental figure S7I), thereby suggesting that its efficacy was at least in part mediated by the increased infiltration of T cells.

## Discussion

Genome-wide analyses of PDAC tissues have evidenced the dysregulation of the axon guidance pathway in this dismal disease.^14 16 55 56^ Here, we investigated the role of the diffusible axon guidance cue SEMA3A, whose tissue expression has been previously linked to poor clinical outcomes in PDAC.^14 15^ We showed that SEMA3A is highly expressed by neoplastic cells with squamous differentiation and a basal-like phenotype. Of the two PDAC epithelial cell lineages,^6–9^ the basal-like/squamous phenotype displays a more aggressive behaviour and it is enriched in post-treatment tumours as well as in metastases.^7^ We found that both cell-intrinsic (eg, biallelic inactivation of p53) and cell extrinsic (eg, TGF-β1) factors promoting the basal-like/squamous subtype induce expression of SEMA3A in PDAC cells. Mechanistically, we demonstrated that SEMA3A exerts both cell-autonomous and non-cell autonomous effects to support the progression of PDAC. Cell-intrinsically, SEMA3A contributes to define a mesenchymal-like phenotype, including enhanced migratory capability. Moreover, tumour-derived SEMA3A activates FAK through the canonical SEMA3A-NRP1 axis to promote anoikis resistance. In keeping with that, SEMA3A overexpressing mouse PDAC cells display superior metastatic competence compared with cells lacking SEMA3A. Moreover, SEMA3A expressing cells induces protumourigenic changes in the TME with increased density of macrophages and significantly reduced infiltration of T cells.

TAMs are the most abundant leucocyte population in the stroma of both mouse and human PDAC^10^ and they contribute to establish an ‘immunologically cold’ microenvironment also through T cell exclusion.^57^ Specifically, in the context of SEMA3A expressing tumours, the depletion of macrophages led to increased intratumoural infiltration of T cells and the maximisation of therapeutic benefit from gemcitabine. The axon guidance is a highly conserved pathway involved in the proper formation of neural circuits during the development of the central nervous system (CNS).^52^ The axon guidance genes include membrane-bound or diffusible ligands (Netrins, Semaphorins, Ephrins, Slits) that act either as chemoattractant or chemorepellent for growing axons and migrating neurons. These axon guidance cues and their receptors are also expressed outside of the CNS where they regulate cell-to-cell, cell-to-extracellular matrix interactions and tissue morphogenesis.^58^ At the molecular level, all guidance cues influence cell motility through the engagement of the Rac family of small GTPases.^58^

Here, we found that SEMA3A sustains gene programmes related to EMT and increases FAK signalling in mouse PDAC cells. The activation of those molecular pathways parallels a functional phenotype of mesenchymal-like cells with migratory capability and increased metastatic competence. Most of the previous studies in PDAC have focused on investigating the role of members of the Slit/Robo axis on the PDAC malignant traits of PDAC as well as its cell identity.^17 19–22^ Of the four classes of ligands, semaphorins represent the largest family and were originally identified as chemorepellent proteins in the nervous system.^23 24^ SEMA3A belongs to the class 3 of secreted semaphorins and its potential role in cancer still needs to be elucidated. Indeed, several works have proposed a tumour suppressive role for SEMA3A, which has been reported to restrain tumour growth by hampering tumour angiogenesis.^59^ In PDAC, an NRP1-independent superagonist SEMA3A was used as vasculature normalising agent which demonstrated antitumour activity.^60^ Moreover, there are contradictory results on the effect of SEMA3A on recruitment and activation of TAMs. TAMs have an established protumoural function and shares features with M2-like macrophages, including the expression of Arginase 1 and of the Mannose Receptor CD206.^61 62^ Carrer *et al* reported that SEMA3A recruits a subset of resident Nrp1+antitumoural macrophages,^63^ while Casazza *et al* found that SEMA3A entraps protumoural macrophages in highly hypoxic areas.^64^ Finally, Wallerius *et al* reported a differential effect of SEMA3A on the proliferation of M2 and M1-like macrophages^65^: SEMA3A favoured the expansion of antitumoural M1-like macrophages which was associated with the recruitment of cytotoxic T cells and a tumour-inhibiting effect.

In our preclinical models, tumour cells derived SEMA3A contributed to define an immunosuppressive TME with abundant macrophages and reduced density of CD8+T cells. Our findings perfectly align with the elevated expression of *SEMA3A* in basal-like/squamous PDAC, which are characterised by elevated infiltration of TAM and scant T cells.^10^ In vitro, SEMA3A functioned as chemoattractant for different macrophages subsets and further skewed the macrophage population towards an M2-like phenotype. Accordingly, the depletion of macrophages with the monoclonal antibody against CSF1R favoured intratumoural infiltration of cytotoxic T cells specifically in the context of SEMA3A^high^ tumours. Nonetheless, we cannot exclude that the establishment of an immunosuppressive microenvironmental contexture in SEMA3A^high^ tumours is also contributed by stromal-derived SEMA3A as well as by SEMA3A-induced neural plasticity.^66^ Furthermore, we found that SEMA3A^high^ tumours were more resistant to gemcitabine treatment than the SEMA3A^low^ tumours, which perfectly aligns with the more aggressive biological behaviour of SEMA3A^high^ tumours. However, the depletion of macrophages resulted in a significant greater benefit in terms of overall survival following chemotherapy for tumours with high expression of SEMA3A.

Overall, we show here that SEMA3A is a functional marker of aggressive PDAC that promotes tumour progression through modification of the local microenvironment and by enhancing the metastatic competence of neoplastic cells. However, a greater infiltration of CD8+T cells is observed in SEMA3A^high^ tumours on macrophages depletion, suggesting a potential chemoattractant role of SEMA3A for T cells. While this aspect needs further elucidation, we have provided proof that the disease control in the setting of SEMA3A^high^ tumours is critically dependent on CD8+T cells. In conclusion, we show that *SEMA3A* is a stress-sensitive locus that enhances the malignant phenotype of PDAC cells through both cell-intrinsic and cell-extrinsic mechanisms.

## Data Availability

All data relevant to the study are included in the article or uploaded as online supplemental information. Data will be made available following acceptance.
