# Supplementary figures and images for: Axon guidance cue SEMA3A promotes the aggressive phenotype of basal-like PDAC
Source: Gut. 2024 Apr 26;73(8):1321–35. doi: 10.1136/gutjnl-2023-329807 (PMC11287654; doi:10.1136/gutjnl-2023-329807)

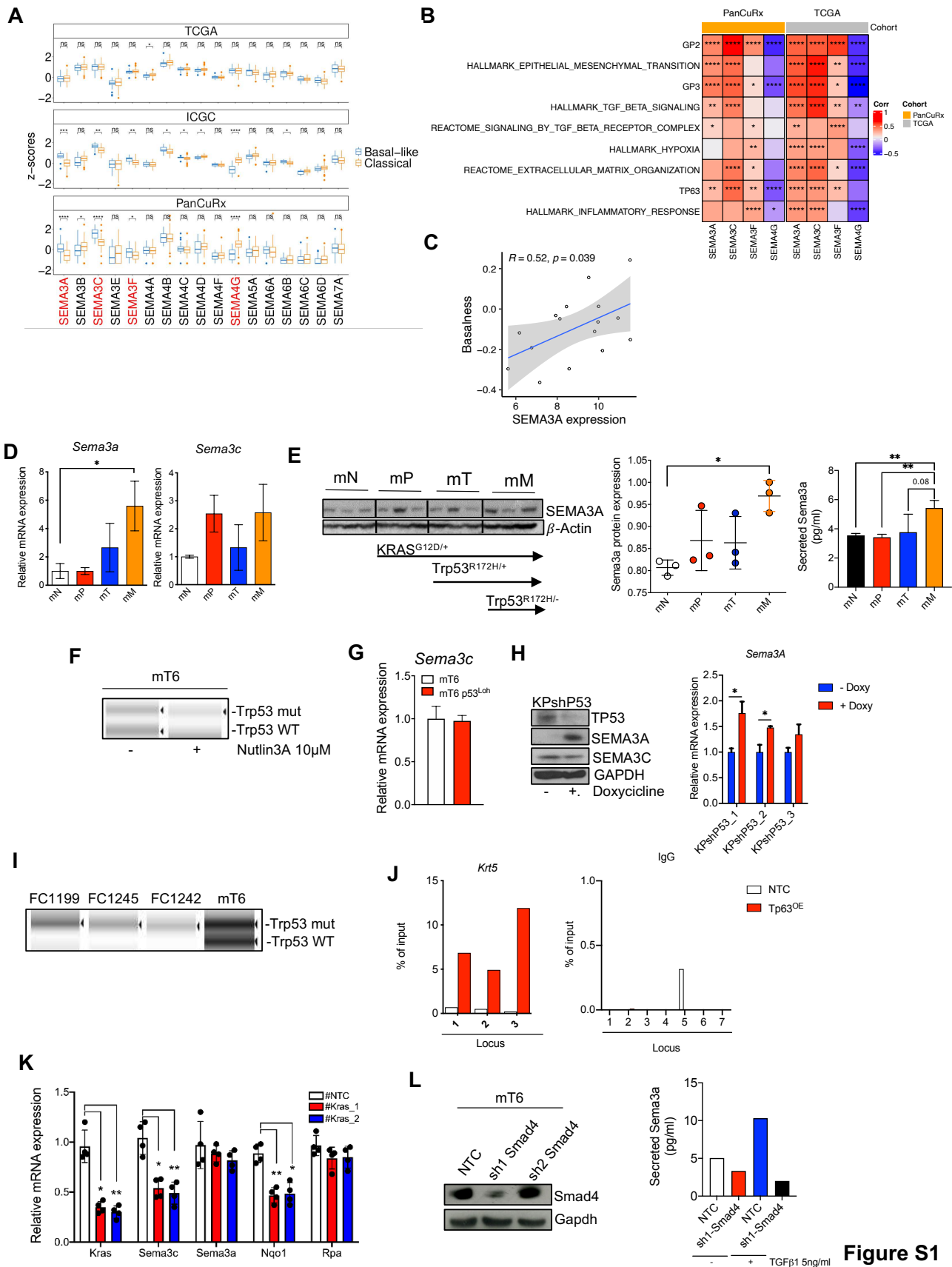

Figure S1

Supplement: Supplementary data [file gutjnl-2023-329807supp001.pdf]

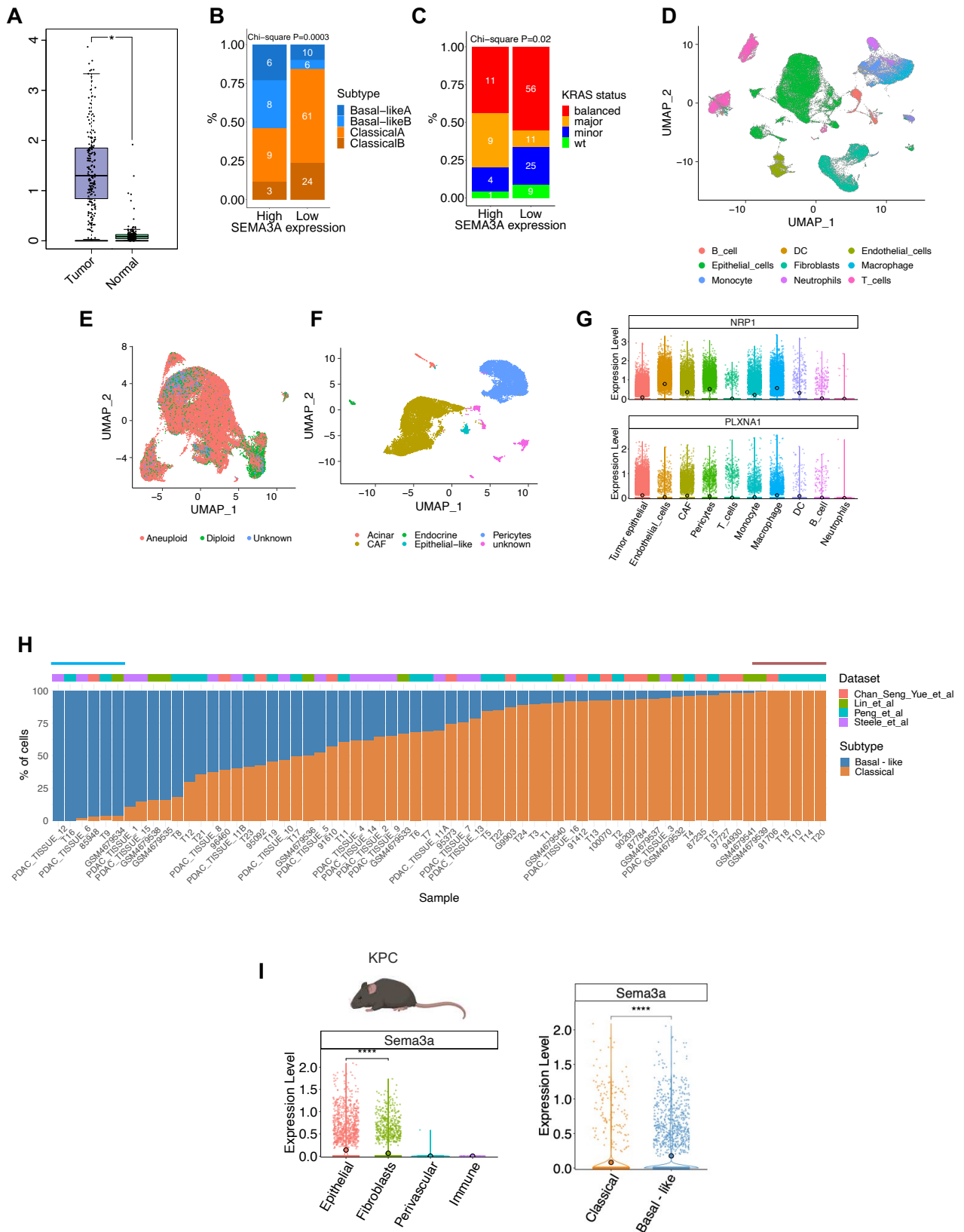

Figure S2

Supplement: Supplementary data [file gutjnl-2023-329807supp002.pdf]

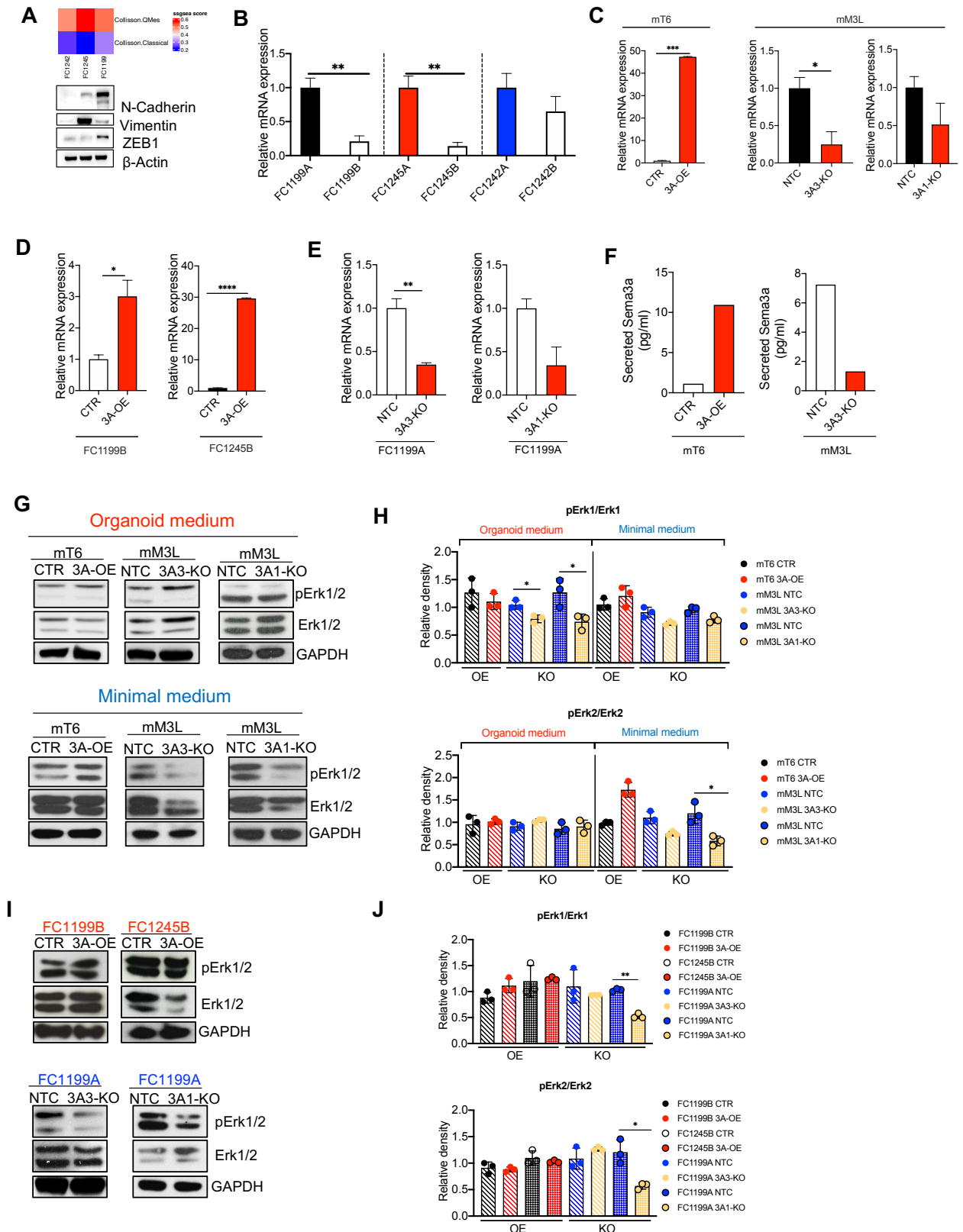

Figure S4

Supplement: Supplementary data [file gutjnl-2023-329807supp005.pdf]

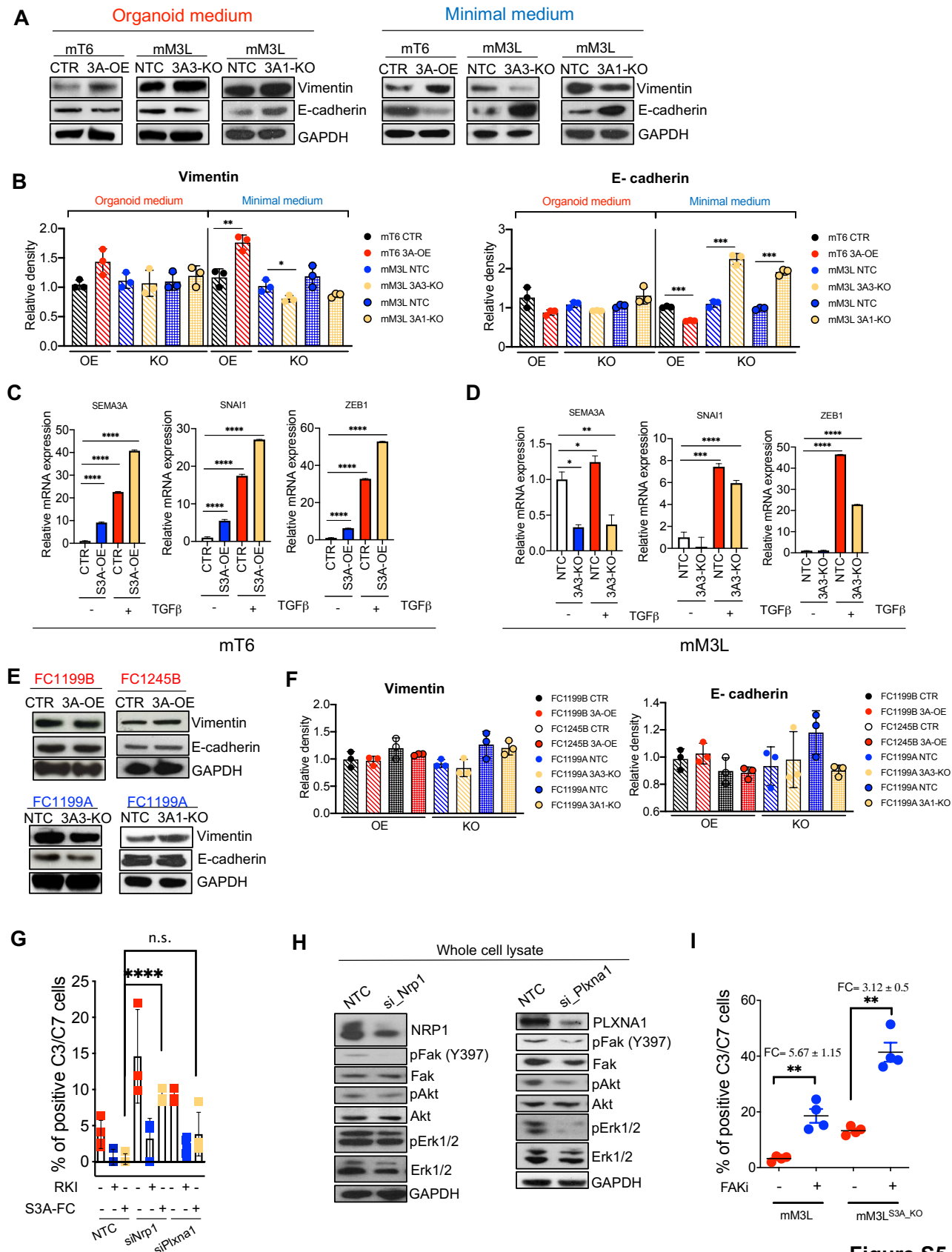

Figure S5

Supplement: Supplementary data [file gutjnl-2023-329807supp006.pdf]

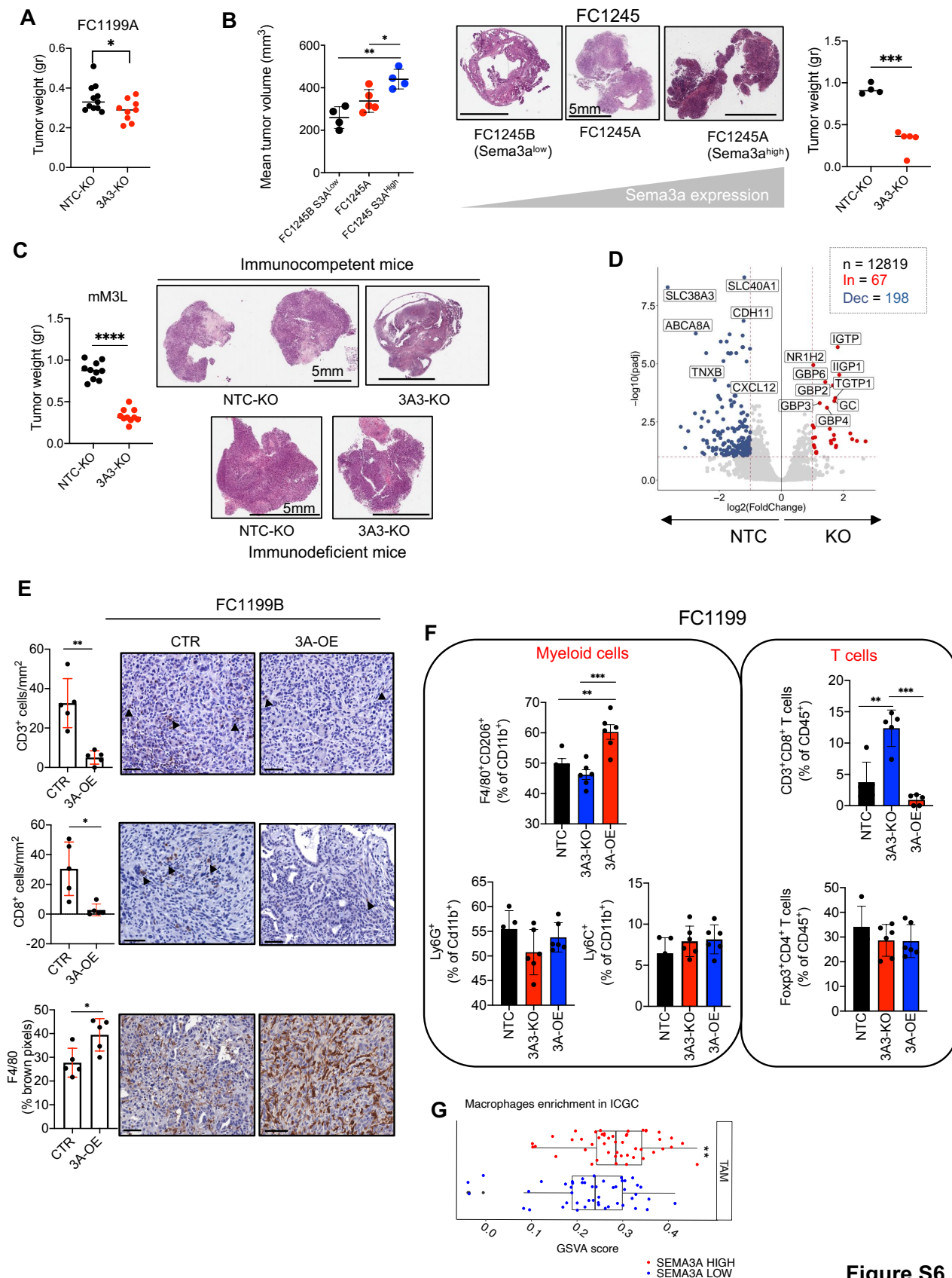

Figure S6

Supplement: Supplementary data [file gutjnl-2023-329807supp007.pdf]

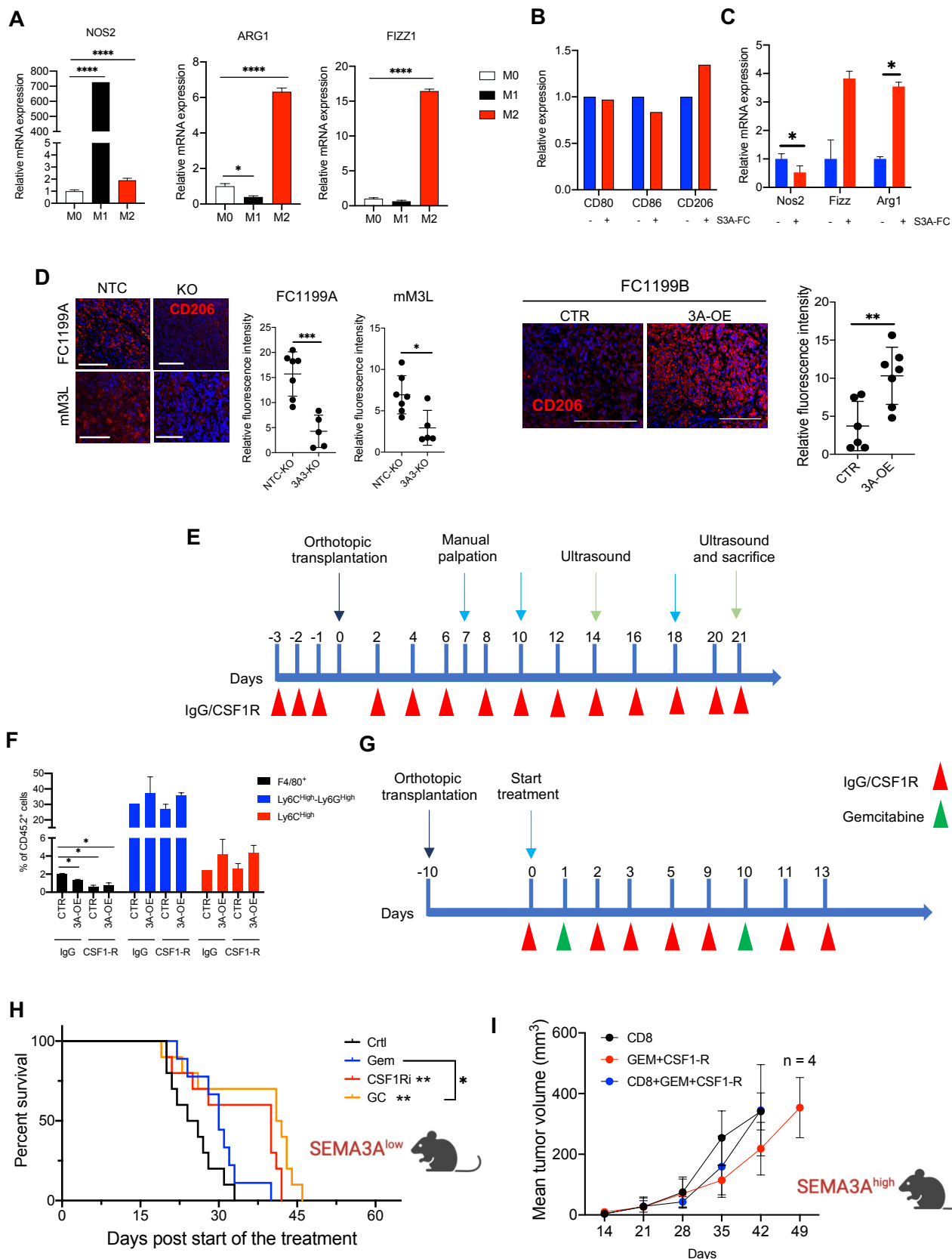

Figure S7

Supplement: Supplementary data [file gutjnl-2023-329807supp008.pdf]
