# Supplementary material for: Axon guidance cue SEMA3A promotes the aggressive phenotype of basal-like PDAC
Source: Gut. 2024 Apr 26;73(8):1321–35. doi: 10.1136/gutjnl-2023-329807 (PMC11287654; doi:10.1136/gutjnl-2023-329807)
Supplement: Supplementary data [file gutjnl-2023-329807supp003.pdf]

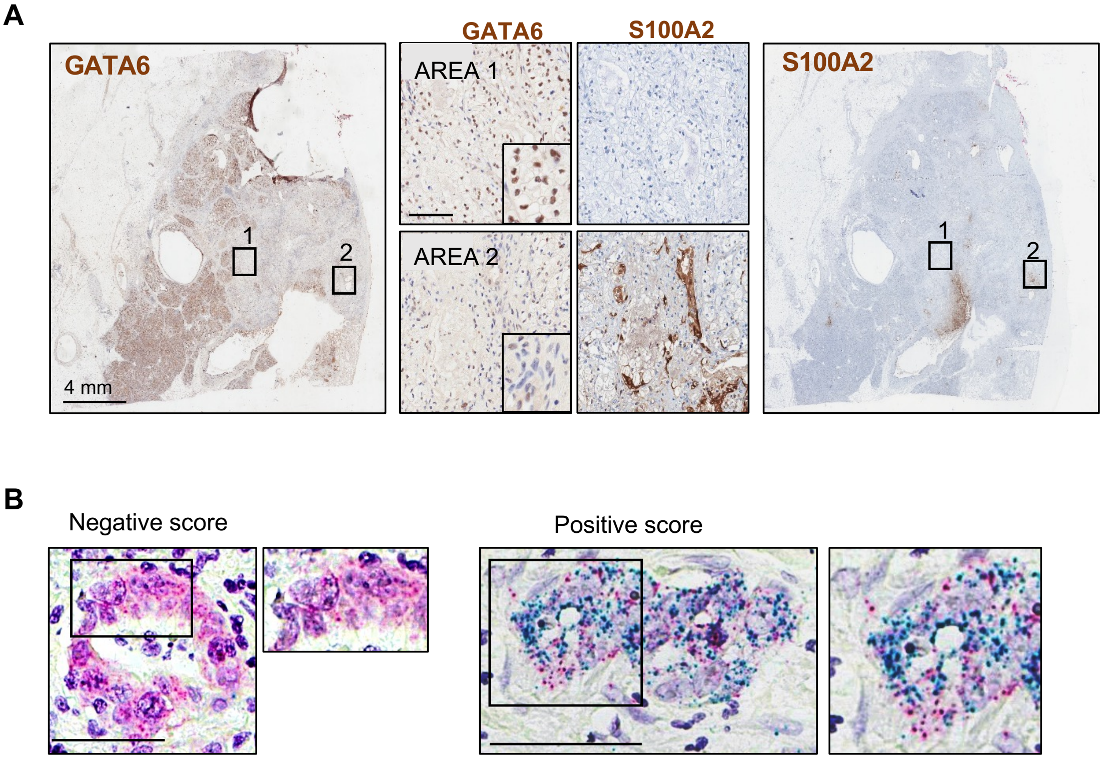


| Staining score | Microscope objective scoring |
|---|---|
| 0 | No staining or less than 1 dot in every 10 cells (40X magnification) |
| 1 | 1-3 dots/cell (visible at 20-40X magnification) |
| 2 | 4-10 dots/cell. Very few dot clusters (visible at 20-40X magnification) |
| 3 | > 10 dots/cell. Less than 10% positive cells have dot clusters (visible at 20X magnification) |
| 4 | > 10 dots/cell. More than 10% positive cells have dot clusters (visible at 20X magnification) |

**Figure S3**
